# Metagenomic analysis reveals associations between salivary microbiota and body composition in early childhood

**DOI:** 10.1038/s41598-022-14668-y

**Published:** 2022-07-29

**Authors:** Modupe O. Coker, Rebecca M. Lebeaux, Anne G. Hoen, Yuka Moroishi, Diane Gilbert-Diamond, Erika F. Dade, Thomas J. Palys, Juliette C. Madan, Margaret R. Karagas

**Affiliations:** 1 Department of Epidemiology, Geisel School of Medicine at Dartmouth College, Hanover, NH 03755 USA; 2 Department of Oral Biology, School of Dental Medicine, Rutgers, The State University of New Jersey, Newark, NJ 07103 USA; 3 Departments of Pediatrics and Psychiatry, Children’s Hospital at Dartmouth, Lebanon, NH 03766 USA

**Keywords:** Medical research, Epidemiology

## Abstract

Several studies have shown that body mass index is strongly associated with differences in gut microbiota, but the relationship between body weight and oral microbiota is less clear especially in young children. We aimed to evaluate if there is an association between child growth and the saliva microbiome. We hypothesized that associations between growth and the saliva microbiome would be moderate, similarly to the association between growth and the gut microbiome. For 236 toddlers participating in the New Hampshire Birth Cohort Study, we characterized the association between multiple longitudinal anthropometric measures of body height, body weight and body mass. Body Mass Index (BMI) z-scores were calculated, and dual-energy x-ray absorptiometry (DXA) was used to estimate body composition. Shotgun metagenomic sequencing of saliva samples was performed to taxonomically and functionally profile the oral microbiome. We found that within-sample diversity was inversely related to body mass measurements while community composition was not associated. Although the magnitude of associations were small, some taxa were consistently associated with growth and modified by sex. Certain taxa were associated with decreased weight or growth (including *Actinomyces odontolyticus* and *Prevotella melaninogenica*) or increased growth (such as *Streptococcus mitis* and *Corynebacterium matruchotii*) across anthropometric measures. Further exploration of the functional significance of this relationship will enhance our understanding of the intersection between weight gain, microbiota, and energy metabolism and the potential role of these relationships on the onset of obesity-associated diseases in later life.

## Introduction

Obesity in young children is associated with premature death and disability in adulthood. In the United States, the prevalence of obesity among children aged 2–5 years increased from 5% in 1980 to over 13% in 2018^1^, making it a significant and growing public health problem. Overwhelming evidence from animal and human studies suggests that the gut microbiome influences the risk of overweight and obesity^2–4^. Several studies (mostly in animals and several in humans) observed differences of important bacterial species in the gut microbiota between obese and normal weight/lean subjects, with some studies showing a higher Firmicutes/Bacteroidetes (F/B) ratio in obese/overweight subjects compared to those of normal weight^5,6^. Data also suggest that lifestyle alterations and physical activity in turn alter the gut microbiome, and that these changes are dependent on obesity status^3,7^. The mechanisms posited to underly these relationships are increased energy harvest, regulation of host metabolism, and the activation of innate immunity.

The relationship between the oral bacteriome and obesity is less clear with emerging evidence suggesting that dysbiosis of the oral microbiome is related to the underlying imbalances and metabolic processes leading to the acquisition of body fat/weight^8,9^. Beyond the well-known orodental diseases like caries, gingivitis and periodontitis, the oral microbiome is associated with systemic inflammation and increased risk for health outcomes including cardiovascular disease, diabetes, rheumatoid arthritis and inflammatory bowel disease^10,11^. As the start of the alimentary canal and home to volumes of saliva ingested daily, the oral cavity has the potential to provide microbial information about the gastrointestinal tract with bacteria regularly passing through the oral cavity to the gut. As observed in the gut, an altered oral microbiome has been associated with metabolic changes and obesity^12–14^ in both adolescents and adults^15^. However, there is limited data on the relationship between obesity and the oral microbiota in early childhood.

In a previous study that compared adults and adolescents with obesity vs. normal weight, body mass index (BMI) was shown to differ significantly with respect to the proportion of *Campylobacter rectus* and *Neisseria mucosa*, as well as *Tannerella forsythia* in the subgingival biofilm with greater abundance^13^. Taxa within the genera *Bifidobacterium,* specifically *B. longum*, and *Lactobacillus* in saliva were cross-sectionally associated with lower obesity prevalence, lower BMI, and lower weight gain. These differences in the microbial composition indicate that there may be distinct patterns of association between the salivary microbiome and obesity.

The oral microbiome is crucial to a child’s oral and systemic health through its role in immune training and seeding of the infant gut^16^. Our focus on early childhood is based on documented changes with complete primary dentition, suggesting establishment of a complex oral microbiome that likely lays a foundation of health with increasing age^17,18^, highlighting the critical need to characterize factors that contribute to the development of the microbiome in early life. Equally important is the likelihood that the oral microbiota mirrors the maturation and stability of the gut microbial community by age 3 observed by several groups^19–21^. High BMI in preschool years (and not later in childhood) has been shown to be associated with a higher risk of overweight or obesity in adolescence among children who had had stable BMI^22^. Therefore, in examining the salivary microbiota of approximately 4-year-old children enrolled in an ongoing prospective cohort, the New Hampshire Birth Cohort (NHBCS)^23–28^, we aimed to investigate whether the salivary microbiota was associated with concurrent body weight metrics (overweight status or body fat mass). For a subset of participating children, we examined the potential impact of early growth exposure metrics prior to sample collection (weight trajectory up to age 2) on the salivary microbiota. Based on existing literature, we hypothesize that salivary microbiota in children would vary in diversity and richness by age, growth scores, adiposity and fat mass.

## Results

### Characteristics of the study population

Out of 273 children enrolled in the NHBCS with saliva microbiome samples available, we focused on 236 that had weight, height, and dual-energy X-ray absorptiometry (DXA) measurements collected at the same time. Of these, the average age of participants was 1410 days (81.9 SD) or almost 4 years old. The distribution of descriptive characteristics for study participants by overweight status is shown in Table 1. The majority (165; 69.9%) of participants were at a normal weight while 62 (26.3%) were classified as overweight or obese. As depicted in Table S1, among the 236 children, 131 (55.5%) were male. To incorporate prospective anthropometric growth data prior to the time of saliva sample collection, we created two exploratory sub-cohorts from the 273 children (Figure S1A) with 137 children conserved in all three sub-populations (Figure S1B). A total of 195 children had weight-for-length/height ratios available (Table S2). Rapid weight gain (RWG), a commonly used dichotomous child growth metric that typically is defined as *an increase in weight-for-age z-score* > *0.67 between birth and a 2-year weight measurement*^29,30^, was assessed in 157 children with available data in the first 2 years of life. Among them, 45 (28.7%) were classified as having RWG (Table S3).

**Table 1:** Descriptive overview of children with BMI, DXA, and saliva microbiome samples at 3–4 years of age by BMI percentile-based groups.

	Underweight	Normal weight	Overweight	Obese	Overall
**BMI percentile**	** < 5th percentile**	**5th to 85th percentile**	**85th to 95th percentile**	$\geq$ **95th percentile**	
**Number of children by group**	**9 (3.8%)**	**165 (69.9%)**	**35 (14.8%)**	**27 (11.4%)**	**236 (100%)**
**Sample age of saliva sample (days)**					
Mean (SD)	1420 (87.4)	1410 (82.3)	1400 (64.1)	1430 (98.2)	1410 (81.9)
Median [Min, Max]	1390 [1320, 1600]	1390 [1160, 1750]	1400 [1310, 1550]	1410 [1310, 1680]	1390 [1160, 1750]
**Sex**					
Male	6 (66.7%)	90 (54.5%)	16 (45.7%)	19 (70.4%)	131 (55.5%)
Female	3 (33.3%)	75 (45.5%)	19 (54.3%)	8 (29.6%)	105 (44.5%)
**Maternal BMI (kg/m**^**2**^**)**					
Mean (SD)	21.9 (1.92)	25.4 (5.02)	26.5 (5.35)	29.0 (6.46)	25.8 (5.33)
Median [Min, Max]	22.4 [18.3, 24.4]	24.1 [17.5, 45.7]	25.4 [18.7, 41.5]	27.0 [21.5, 45.2]	24.4 [17.5, 45.7]
Missing	0 (0%)	4 (2.4%)	2 (5.7%)	0 (0%)	6 (2.5%)
**Delivery method**					
Vaginal	8 (88.9%)	120 (72.7%)	23 (65.7%)	19 (70.4%)	170 (72.0%)
C-section	1 (11.1%)	45 (27.3%)	11 (31.4%)	8 (29.6%)	65 (27.5%)
Missing	0 (0%)	0 (0%)	1 (2.9%)	0 (0%)	1 (0.4%)
**Gestational age at birth (weeks)**					
Mean (SD)	38.5 (1.24)	39.0 (1.83)	38.9 (2.27)	38.9 (1.88)	39.0 (1.88)
Median [Min, Max]	38.6 [36.7, 40.0]	39.3 [31.6, 42.0]	39.0 [31.0, 43.0]	39.3 [34.3, 41.4]	39.1 [31.0, 43.0]
**Solid foods start age (months)**					
Mean (SD)	5.44 (1.59)	5.36 (1.28)	5.07 (1.33)	4.92 (1.22)	5.28 (1.30)
Median [Min, Max]	6.00 [3.00, 8.00]	6.00 [1.00, 10.0]	5.00 [3.00, 8.00]	5.00 [3.00, 8.00]	5.00 [1.00, 10.0]
Missing	0 (0%)	19 (11.5%)	6 (17.1%)	5 (18.5%)	30 (12.7%)
**Body Mass Index (kg/m**^**2**^**)**					
Mean (SD)	13.5 (0.256)	15.6 (0.762)	17.4 (0.301)	18.8 (0.716)	16.2 (1.40)
Median [Min, Max]	13.6 [13.1, 13.9]	15.7 [13.7, 17.0]	17.3 [16.9, 18.0]	18.8 [17.9, 21.3]	16.0 [13.1, 21.3]
**Height (cm)**					
Mean (SD)	100 (4.50)	102 (4.18)	102 (3.42)	104 (4.84)	102 (4.26)
Median [Min, Max]	98.3 [92.9, 106]	102 [91.2, 117]	101 [95.7, 109]	104 [96.2, 115]	102 [91.2, 117]
**Weight (kg)**					
Mean (SD)	13.5 (1.11)	16.2 (1.61)	18.0 (1.26)	20.6 (2.08)	16.8 (2.26)
Median [Min, Max]	13.1 [12.0, 15.1]	16.2 [12.9, 19.6]	17.9 [15.5, 21.1]	20.4 [16.7, 25.0]	16.7 [12.0, 25.0]
**Total fat mass (g)**					
Mean (SD)	3290 (644)	4920 (898)	6200 (989)	7370 (1350)	5330 (1340)
Median [Min, Max]	3340 [1970, 3990]	5000 [2550, 7430]	6010 [3980, 7940]	7330 [5050, 10500]	5200 [1970, 10500]
**Total lean mass (g)**					
Mean (SD)	9860 (1100)	10,900 (1300)	11,400 (1430)	12,800 (1840)	11,200 (1530)
Median [Min, Max]	9500 [8250, 11500]	10,900 [8090, 14600]	11,100 [8900, 15500]	12,400 [9940, 16900]	11,100 [8090, 16900]

DXA scanning provided values for total fat mass (TFM) and total lean mass (TLM) in grams. Ultimately, five variables were selected to assess body composition for children at 3–4 years of age: (1) TFM as reported from DXA; (2) TLM as reported from DXA; (3) age- and sex-adjusted BMI z-score; (4) age- and sex-adjusted BMI percentiles to categorize children as underweight (< 5th), healthy weight (5th to < 85th), overweight (85th to < 95th), or obese (≥ 95th); and (5) a binary variable for overweight with the age- and sex-adjusted BMI 85th percentile used as the threshold.

### Relationships between child growth measurements

**Figure 1:**
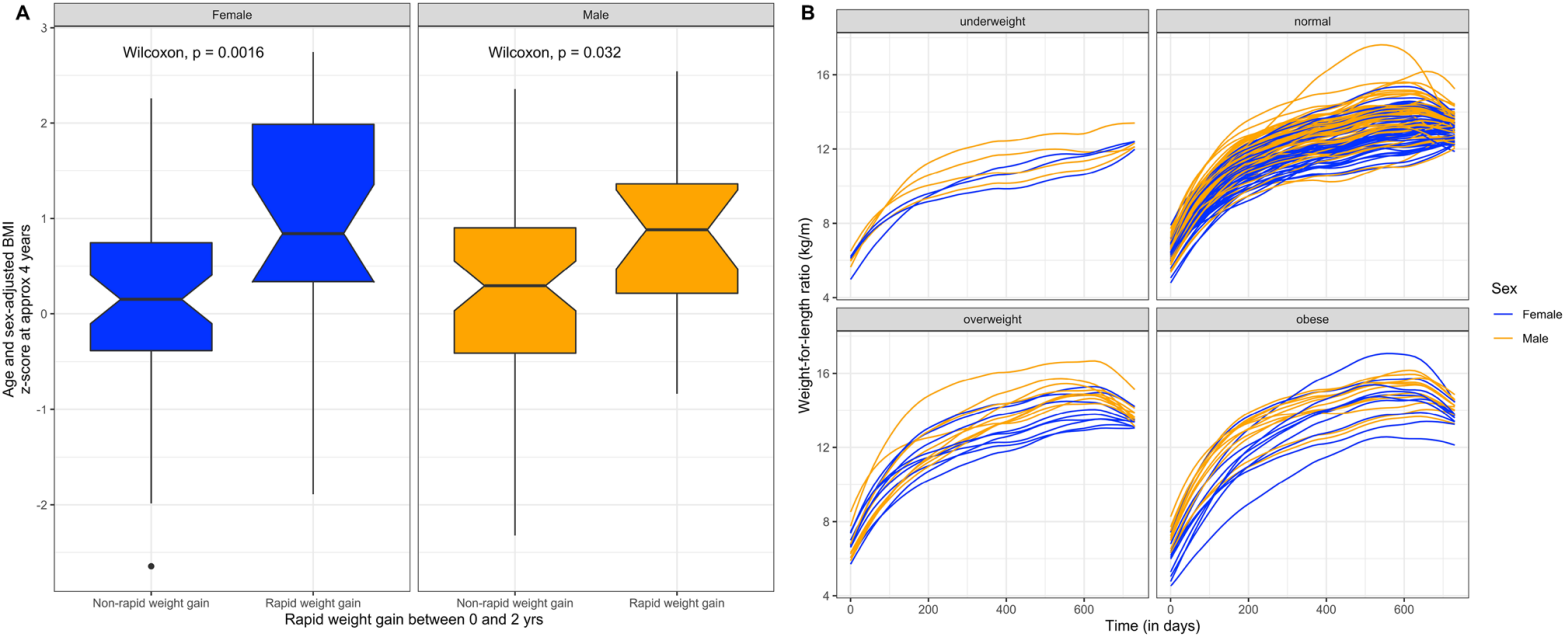
Associations between body mass index (BMI) measured at 3 or 4 years of age versus child growth metrics measured between 0 and 2 years of age. (**A**) Age and sex adjusted BMI z-score versus rapid weight gain stratified by sex for 157 children. Wilcoxon p-value indicates difference in BMI z-score by rapid weight gain group for females and males. (**B**) Growth charts using the weight-for-length (weight-for-height) ratio plotted for 195 children between the ages of 0 and 2 stratified by BMI percentile groupings at 3 or 4 years of age. Growth indices are colored by the sex of the child.

Body mass index and DXA-measured TFM were highly but not perfectly correlated (Pearson correlation, r = 0.80) (Figure S2A), whereas DXA-measured lean mass was only moderately correlated with BMI (r = 0.47) (Figure S2B). Likewise, in linear regression models, adjusted for age and sex, a unit BMI increase was associated with a 777 (95% CI: 705, 848) gram increase in TFM and a 499 (95% CI: 392, 607) gram increase in TLM respectively. As expected, male and female children had different mean anthropometric measurements with male children being taller (Kruskal–Wallis p < 0.05), heavier (p < 0.05), and with a higher amount of TLM (p < 0.05). Using our exploratory sub-cohorts, we validated that both rapid weight gain between 0 and 2 years and weight-for-length ratio were associated with BMI at 3–4 years of age among both males and females (Fig. 1A and Fig. 1B). Rapid weight gain was defined as a > 0.67 increase in weight-for-age z-score between 0 and 2 years of age with 0.67 indicative of the difference between percentile bands measured on a standardized growth chart.

### Community composition of salivary microbiota by growth status

**Figure 2:**
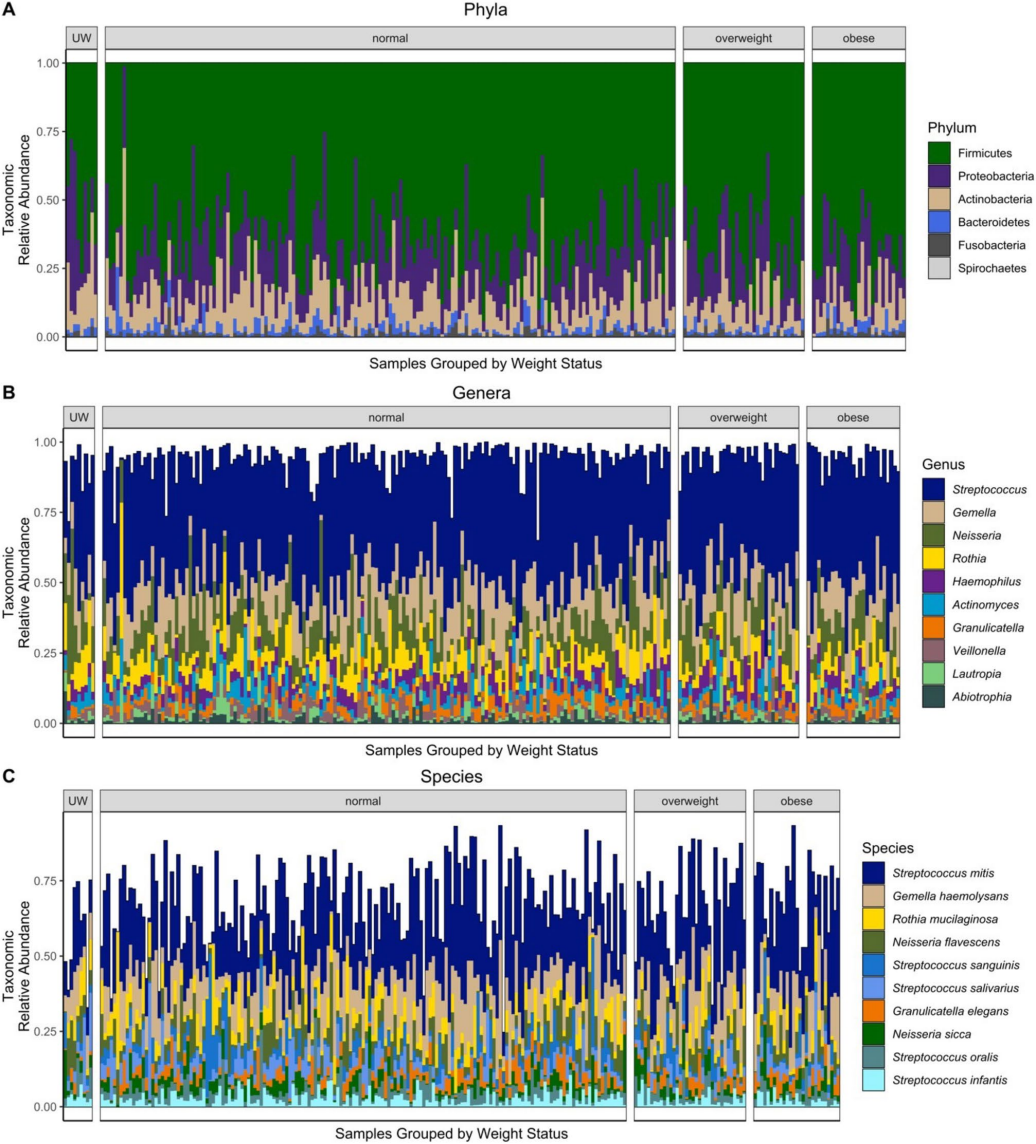
Saliva microbiome composition among 236 children grouped by body mass index percentile groupings. (**A**) Phylum-specific composition by mean relative abundance. (**B**) The composition of the top 10 genera based on the highest mean relative abundance across all samples. (**C**) The composition of the top 10 species based on the highest mean relative abundance across all samples. Color ranges (i.e., spectrum of blue for *Streptococcus*) for species are used to delineate species from the same genus. UW = underweight.

A total of 627 GB of raw data was generated from the Illumina NextSeq platform. After filtering out low-quality data and host contamination, an average of 22.9 million reads of clean data were retained for each sample. The majority of saliva microbiota was made up of Firmicutes followed by Proteobacteria, Actinobacteria, and Bacteroidetes (Fig. 2A). From the 236 children, 203 species and 54 genera were prevalent at an abundance of 1% across all samples. The top genera by mean relative abundance (Fig. 2B) were *Streptococcus*, *Gemella*, and *Neisseria* and the top species (Fig. 2C) were *Streptococcus mitis, Gemella haemolysans,* and *Rothia mucilaginosa*. Limited differences in the relative abundance of species were noted by weight status group, but samples coming from underweight children showed the most variation from the other groups with a much lower abundance of *Streptococcus mitis* compared to the other groups (13.5% compared to 23.9–28.5%) (Table S4).

**Figure 3:**
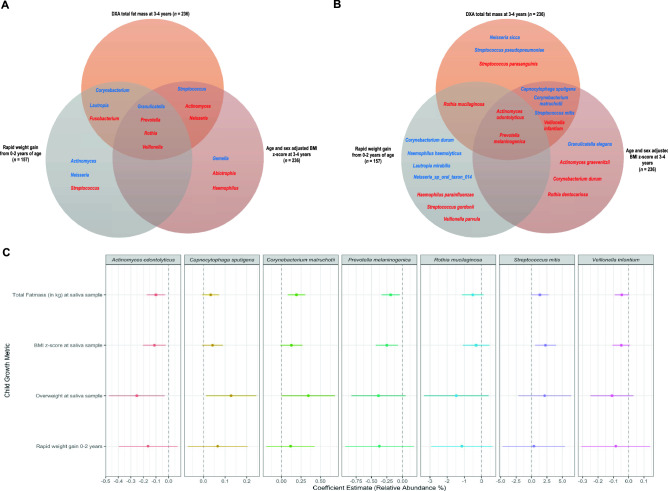
Comparison of associations between growth metrics and microbes. (**A**) Venn diagram depicting the top 10 genera by lowest p-value produced from adjusted MaAsLin2 regressions. (**B**) Venn diagram depicting the top 10 species by lowest p-value. For both (**A**) and (**B**), blue and red are indicative of positive and negative coefficients respectively. (**C**) Dot and whisker plots to represent the relative abundance change attributable to the child growth metric. Each row represents the coefficient estimate from a different linear regression model. These adjusted regression models included the exposure (growth variable) and the following covariates: delivery mode (vaginal or cesarean), sex (male or female), sample age in days, maternal BMI, gestational age in weeks, and solid foods start age in months. The sample size of the adjusted models for the growth metrics measured at the time of saliva sample collection was 202. The sample size for the rapid weight gain model was 138. Species were selected for univariate linear regression analysis due to their overlap in the species-level Venn diagram.

Using MaAsLin2, we explored associations between child growth metrics and the relative abundance individual saliva microbiota (Table S5). Although we found limited results reaching statistical significance (Benjamini–Hochberg q-value < 0.25), we observed a high level of concordance in the direction of associations for taxa across metrics assessed. Of the top 10 genera by p-value in adjusted models for TFM and BMI z-score at 3 or 4 years of age, 7 genera overlapped (Fig. 3A). Of those 7, *Granulicatella* and *Streptococcus* abundance were positively associated while *Actinomyces*, *Neisseria*, *Prevotella*, *Rothia*, and *Veillonella* were negatively associated. Regarding species, six species overlapped between growth measurements with *Actinomyces odontolyticus* and *Prevotella melaninogenica* abundance consistent across all three models (Fig. 3B). To further demonstrate the consistency and effect size similarities between models, we subsequently ran adjusted univariate linear regression models on specific taxa and plotted the overlap between species (Fig. 3C). Across all these species with a low p-value via MaAsLin2, the effect estimates across all child growth metrics were consistent but effect sizes were small.

**Figure 4:**
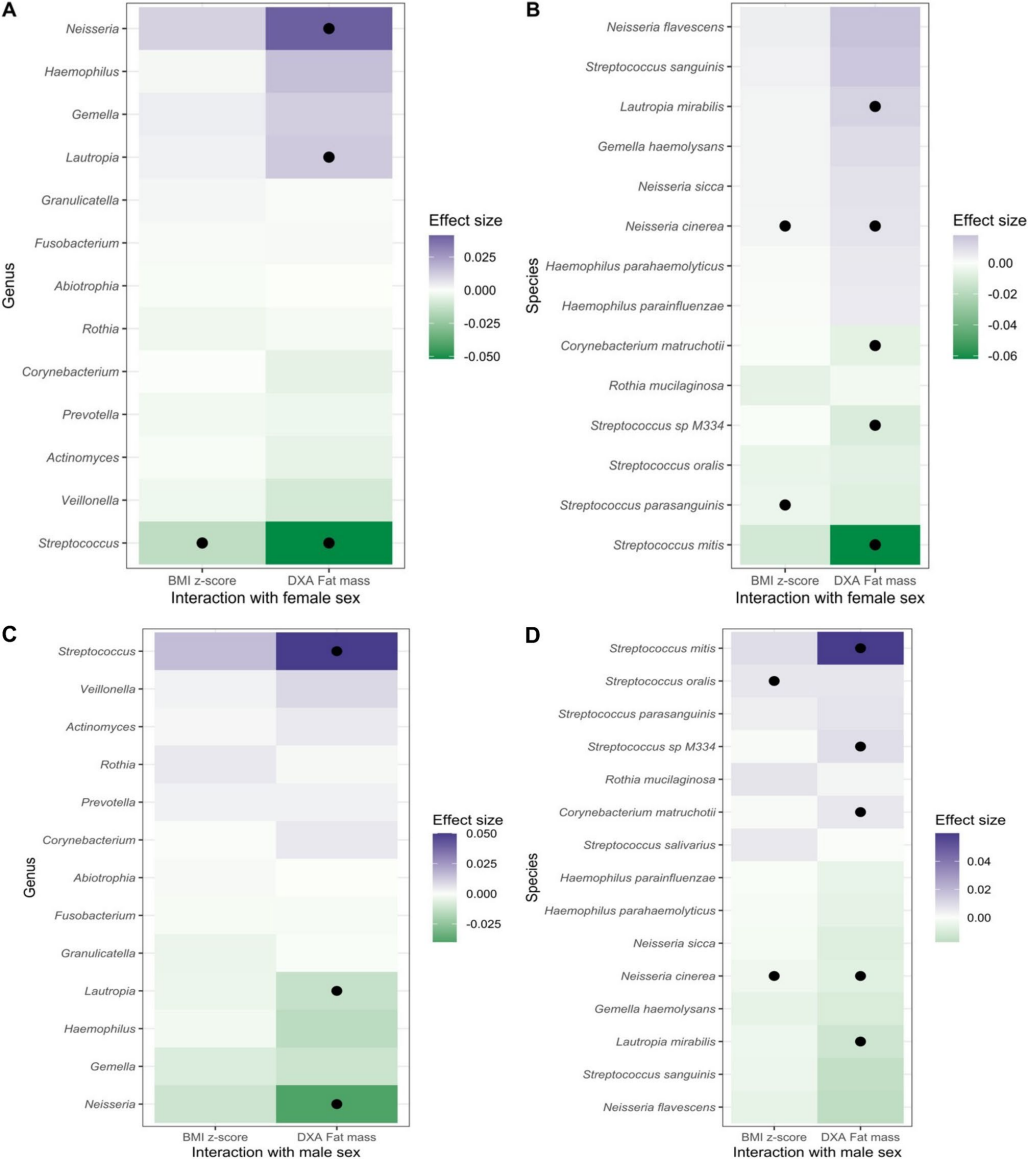
Assessing the joint effects of body mass and sex on saliva microbiota at 3 or 4 years of age. MaAsLin2 models included in addition to the interaction term: the growth variable, sex (male or female), delivery mode (vaginal or cesarean), sample age in days, maternal BMI, gestational age in weeks, and solid foods start age in months. Black circles indicate a p-value < 0.1. (**A**) Coefficient for interaction between female sex and child growth metric (age and sex adjusted BMI z-score or total fat mass in grams using a DXA scan) on the relative abundance of genera. (**B**) Coefficient for the interaction between female sex and child growth metrics on the relative abundance of species. (**C**) and (**D**) use the same data but the models for the interaction term represents joint effects with males instead of females. For species, only associations with an effect size > 0.005 or < − 0.005 are included.

Due to inherent differences in child growth by sex, we were interested in assessing the joint interaction of sex and child growth on the saliva microbiota. At the genus level, we found evidence of an antagonistic joint effect of female sex and child growth on *Streptococcus* while assessing both age and sex-adjusted BMI z-score as well as DXA-measured fat mass (Fig. 4A) At the species-level, we found consistent results to the genus-level analysis. A synergistic joint effect of female sex and child growth on *Neisseria cinerea* was also observed (Fig. 4B). For males, an antagonistic joint effect with child growth was noted for *Neisseria* and *Neisseria cinerea* (Fig. 4C; Fig. 4D)*.*

### Microbial diversity of salivary microbiota by growth status

In addition to community composition, we were interested in associations between body mass and saliva microbiome diversity metrics. Moderate dose-dependent associations between weight status and Shannon alpha diversity were noted; samples from underweight, normal weight, overweight, and obese children had a mean (standard deviation) Shannon diversity metric of 2.84 (0.23), 2.75 (0.45), 2.71 (0.46), 2.68 (0.51) respectively. Alpha diversity measured by the Shannon index showed a moderately inverse association with BMI z-scores (Table 2). TFM was associated negatively with alpha diversity in a crude/unadjusted analysis. TLM showed no significant association (data not shown but the analysis was otherwise identical to TFM).

**Table 2:** Association between Shannon alpha diversity of saliva samples and child growth metrics measured at 3–4 years. Each cell shows the linear regression coefficient for each exposure’s association with the Shannon index as derived from 6 different linear regression models. A crude and adjusted linear regression model was ran for each of our main body mass exposures of interest: total fat mass (in kg), body mass index z-score, and overweight status (with age- and sex-adjusted BMI at the 85th percentile used as the threshold) respectively.

	Dependent variable: Shannon alpha diversity (95% CI)					
	Model coefficients					
	Crude TFM	Crude BMI z-score	Crude overweight status	Adjusted TFM	Adjusted BMI z-score	Adjusted overweight status
Total fat mass in kg	− 0.041* (− 0.084, 0.002)			− 0.034 (− 0.083, 0.015)		
BMI z-score		− 0.056** (− 0.110, − 0.002)			− 0.060* (− 0.119, 0.0001)	
Overweight			− 0.056 (− 0.188, 0.075)			− 0.036 (− 0.183, 0.111)
Solid foods start age (months)				− 0.007 (− 0.058, 0.044)	− 0.008 (− 0.059, 0.043)	− 0.007 (− 0.059, 0.044)
Female				− 0.038 (− 0.166, 0.089)	− 0.045 (− 0.172, 0.081)	− 0.044 (− 0.172, 0.084)
Log_10_-transformed sample age in days				− 1.802 (− 4.413, 0.809)	− 1.82 (− 4.412, 0.771)	− 1.917 (− 4.534, 0.700)
Gestational age (in weeks)				− 0.011 (− 0.045, 0.024)	− 0.011 (− 0.045, 0.023)	− 0.012 (− 0.046, 0.023)
C-section				− 0.022 (− 0.170, 0.125)	− 0.02 (− 0.167, 0.126)	− 0.023 (− 0.171, 0.125)
Maternal BMI (kg/m^2^)				0.015** (0.003, 0.028)	0.017** (0.004, 0.029)	0.015** (0.002, 0.027)
Intercept	2.954** (2.717, 3.191)	2.757** (2.696, 2.818)	2.751** (2.684, 2.819)	8.682** (0.589, 16.774)	8.562** (0.513, 16.611)	8.931** (0.808, 17.054)
Observations	236	236	236	202	202	202
R^2^	0.014	0.017	0.003	0.055	0.064	0.047
Adjusted R^2^	0.01	0.013	− 0.001	0.02	0.03	0.012

p < 0.1 = * and p < 0.05 = **; TFM = total fat mass, BMI = body mass index.

Between-sample or beta diversity analysis using principal coordinate analysis (PCoA) plots showed moderate associations with TFM and BMI z-scores (Figure S3). Unlike TFM and BMI z-scores, overweight status was not statistically significantly associated with beta diversity. Within PERMANOVA models, TFM and BMI z-scores were statistically significant (Table S6 and Table S7; crude model p-values < 0.05; adjusted model p-values < 0.1) with microbial beta diversity but described very little of either model’s variation.

### Exploratory functional analysis of saliva microbiome by growth status

**Figure 5:**
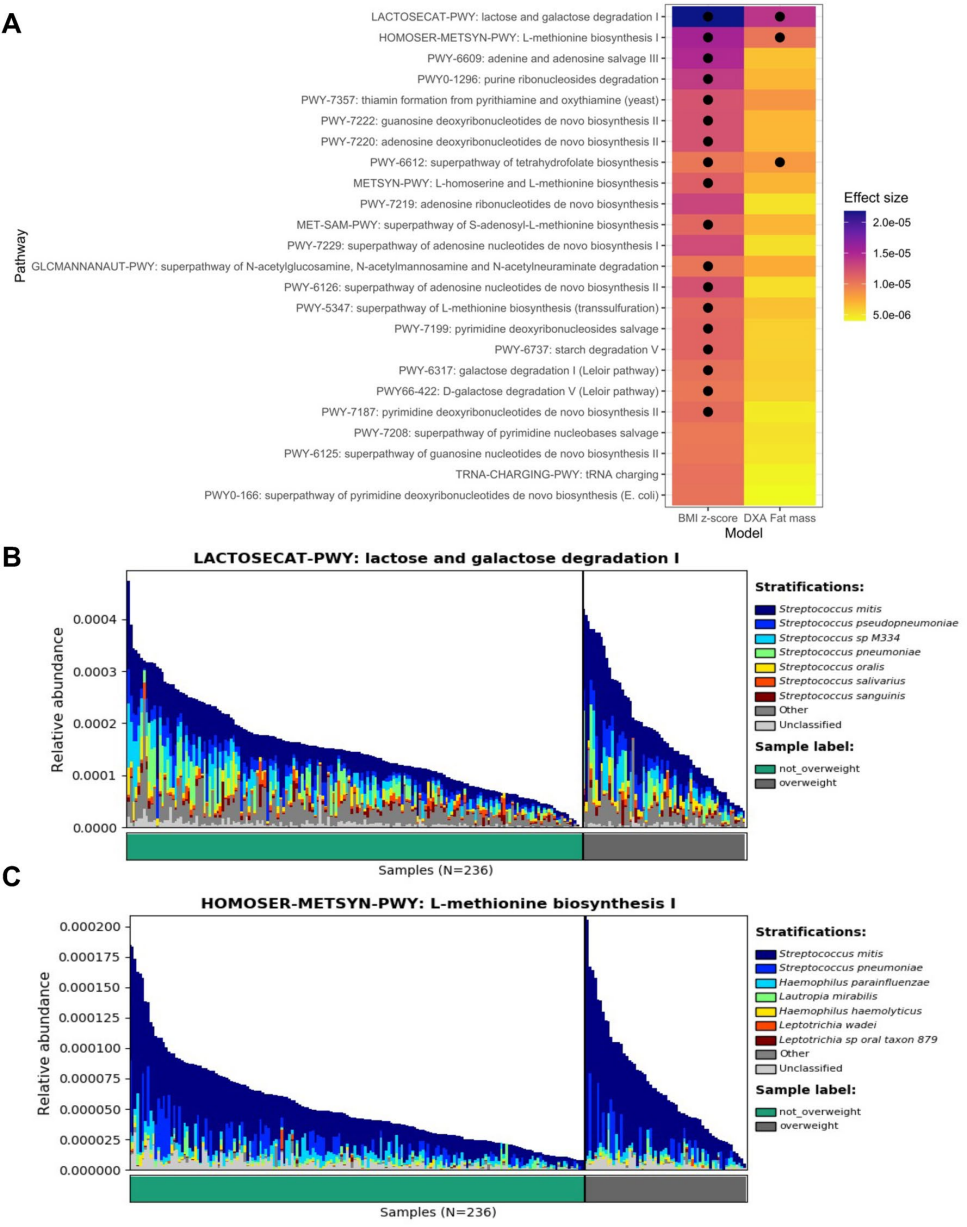
Exploratory analysis of body mass metrics and saliva microbiome functional profiles from pathway analysis. (**A**) Tile plot demonstrating the effect size and statistical significance of associations between child growth metrics (age and sex-adjusted BMI and DXA measured total fat mass in kilograms) and functional pathways. Effect sizes are derived from MaAsLin2 analyses. The models were adjusted for delivery mode (vaginal or cesarean), sex (male or female), sample age in days, maternal BMI, gestational age in weeks, and solid foods start age in months. Black circles represent a *p*-value < 0.15. Only associations with an effect size absolute value greater than 0.00001 in at least one of the two models were included in the plot. (**B**) and (**C**) Species-specific contributions of select pathways and KEGG gene families by overweight status. Pathways shown were selected from results from (**A**) and had the highest effect size in the two models.

In addition to profiling taxa present in the samples, we aimed to better understand how child growth might be associated with functional differences in the saliva microbiome. While results did not indicate strong associations between child growth metrics and pathway abundances, we found consistently positive associations across models assessing TFM and age and sex-adjusted BMI z-scores. Among functional pathways, we identified sugar and methionine pathways including lactose and galactose degradation I and L-methionine biosynthesis I to be statistically significant but with small effect sizes (Fig. 5). *Streptococcus* species (predominantly *S. mitis*) were important in these functional pathways matching and provide further context to how *Streptococcus* may be associated with child growth.

### Associations between other early-life factors and the saliva microbiome

As this study is one of the first to profile oral microbiomes with shotgun metagenomics in children 3–4 years of age, we performed exploratory analyses of the impact of other early-life factors on saliva microbiota. Regarding diversity metrics, we found maternal BMI was positively associated with Shannon alpha diversity in all models (Table 2). Maternal BMI, age of saliva sample collection, and sex were moderately associated with beta diversity but no variables explained a significant portion of the variance (Table S7). Additionally, in our differential abundance analyses using MaAsLin 2 (Table S5), we consistently identified maternal BMI to be positively associated with *Veillonella parvula* and *Veillonella.* Independently, increasing age of the child and female sex were also associated with increased abundance of *Haemophilus*. While other associations between early-life exposures and microbes were often of similar direction and magnitude across models, no other early-life exposures were found to be statistically significant (Benjamini–Hochberg q-value < 0.25).

## Discussion

In the present study, we characterized the oral microbiome using shotgun sequencing technology and investigated its relationship with age- and sex-adjusted BMI and body composition. Our study is among the first to provide comprehensive metagenomic insight into the association between growth outcomes and the salivary microbiome in early childhood. While there were no strong taxa-specific associations, we identified multiple bacterial taxa (including *Actinomyces, Corynebacterium, Capnocytophaga, Prevotella, Streptococcus mitis and Veillonella)* to be moderately associated with TFM, a child’s overweight status, BMI and RWG. Further, we identified high levels of concordance for these taxa with respect to abundance and direction across the various anthropometric measurements. Our study highlights the potential interactions between child growth and sex, with an antagonistic interaction noted for *Streptoccocus* abundance among females but a synergistic interaction with *Neisseria cinerea*. Overall, we found that various taxa within the phylum Firmicutes, Actinobacteria, and Bacteroidetes were associated with body composition and weight gain in the first two years of life.

Bacterial- and host-genome-association studies of obesity are complex, multifactorial and bidirectional in nature. The wide range of host and environmental effects and the significant inter-individual variability of the oral microbiome makes interpretation of studies, such as ours, challenging. Furthermore, in examining the association between oral microbiota and obesity in pre-school children, the accurate assessment of growth and adiposity is critical. We observed that BMI Z-scores were more highly correlated with DXA-derived fat mass compared to lean mass, confirming earlier reports and providing validation to the anthropometric measurements^31^.

Literature provides strong evidence of significant differences in the human gut microbiome comparing people with obesity to controls^2,4^. There is some consensus of increased levels of gut Firmicutes to the detriment of Bacteroidetes^32^ with obesity and type 2 diabetes. Early-life gut microbiota is strongly influenced by dietary factors including the introduction of formula and solid food^21,23,27^. The most dominant and differentially abundant taxa in the infant gut due to obesity was Firmicutes followed by Bacteroidetes^33^*,* as has also been found in adult studies^34^. It has been hypothesized that having higher gut levels of Firmicutes promotes more efficient storage of energy from a given diet among obese subjects compared with lean subjects. Although gut studies focus on fecal bacteria, all bacteria from the gastrointestinal tract must pass through the oral cavity and are potentially seeded from the oral cavity^35^. The relationship between the oral microbiota composition and obesity is less clear as there have been mixed and inconsistent results. This is primarily due to variation in study population, methodology, body weight assessments and microbiome characterization. Our study is among the first to utilize WGS, include more than 100 participants or focus exclusively on pre-school children. Previous studies have reported no differences in oral microbiota composition according to BMI^36,37^ while others have observed distinct features^38–40^. Nevertheless, there is growing evidence of a significant association between levels of specific oral bacterial taxa and obesity, BMI and weight gain^39,41,42^. This increase in attention stems from the relationship between body weight and oral health, specifically the manifestation of periodontitis, gingivitis and dental caries. These findings lead to a logical thread of investigations related to answering the question “Is the relationship between obesity and oral health mediated by the oral microbiome?”.

Our findings of lower diversity in the oral microbiome with increasing BMI and fat mass are in line with previous studies^43^. In contrast to our findings, several studies observed no difference^36^ while one study reported a higher diversity in obese children^44^. We observed no clear clustering of beta diversity indices by BMI or DXA measurements. This finding is likely due to the large inter-individual variation in the salivary microbiome among healthy-weight children leading to considerable overlap in distance measures as observed by others^36^. Our study findings highlight lower levels of *Prevotella* (from phylum Bacteroidetes) in overweight children. Goodson et al.^45^ reported that oral *Prevotella spp*. was more abundant in overweight women compared to normal weight women. In contrast, no significant association between oral Bacteroidetes with obesity was observed in a large study of African-American adults aged > 50^39^. *Prevotella* species dominate in periodontal diseases and abscesses and are often associated with mucosal inflammation^46^. *Prevotella* in the gut has been previously shown to be negatively associated with BMI and fat mass in children^47^ as we observed with saliva. Other studies of adolescents and adults have identified *Prevotella* to be positively associated with aging and pro-inflammatory cytokines^48^, which is consistent with findings that obesity is associated with low-grade inflammation. Overall, these conflicting findings signal the need for future work.

Sex-specific differences in the association between growth and salivary microbiota observed in this study, though mechanism unclear, has been previously reported^49,50^. A study of 483 girls and 417 boys in Finland revealed large, though not significant, gender-dependent body size-related differences in microbial diversity and bacterial abundance^43^. Ortiz et al. also reported significant differences in the salivary microbiome between caries-active and caries-free boys and girls^51^. Gut microbiota studies have reported distinct sex-specific taxonomy and functional phenotypes^24,52–54^ particularly in relation to neurodevelopment^55^. Some prepubescent, adolescent and adult studies have attributed these shifts or differences to the endocrine system^56^, suggesting an interplay between hormones, fat deposition and the microbiota. Study participants for this current study were approximately 4 years of age suggesting that these differences might be in the incipient stages. Additional studies are needed to further elucidate this relationship.

The relationship between periodontal disease and obesity has also drawn more attention to the role of the oral microbiota in obesity. Adult studies have shown that obesity is associated with increased counts and proportions of certain periodontal pathogens, including *Tannerella forsythia* and *Selenomonas noxia*^57^. Our study population offers an advantage of examining the association between oral microbiota and obesity in childhood as children are not typically at risk of inflammatory diseases or conditions associated with aging such as periodontitis, diabetes and cardiovascular disease therefore the associations with obesity can be the focus of investigation^58^. Consistent with this premise, and as expected, we did not observe any significant associations between body weight and well known pathobionts. *S. mitis* (considered one of the beneficial commensal bacteria and an emerging opportunistic pathogen when in niches distal to the oral cavity) was observed as being among the most taxonomically abundant and functionally active species with respect to anthropometric measures. Therefore, the contribution of *S. mitis* to childhood growth and weight/fat gain requires further examination.

RWG in early childhood has been identified as a risk factor for obesity in adolescence and adulthood and its associated complications^59^. We report differentially abundant taxa based on RWG that seemed to overlap with other growth measures. However, we identified two overlapping taxa were associated in opposing directions for RWG compared to concurrent BMI Z scores. It is not clear if these differences are due to distinct periods of childhood growth. Furthermore, RWG in the first 2 years of life was not observed in all children who were overweight at approximately 4 year of age, suggesting that RWG in early life does not always reflect the same growth patterns later in life. Craig et al.^41^ sequenced hypervariable regions V3 and V4 of the 16S rRNA gene in oral and stool samples from over 200 two-year-olds and utilized functional data analysis to examine childhood weight-gain trajectories longitudinally. The authors report that in children who gained weight rapidly from birth to six months of age, oral bacterial diversity at two years of age was decreased with a higher Firmicutes to Bacteroidetes ratio; but this was not observed with the gut microbiota^41^. While within-sample diversity and F/B ratio are key summary tools for assessing of the microbiota, they are limited in their ability to identify obesity-related features of the fecal or salivary microbiota. Nevertheless, the findings from Craig and colleagues^41^ suggest that obesity-related associations may appear at an earlier time point for saliva microbiota than in the gut microbiota. Future investigations may hold promise of leveraging the oral microbiome as a biomarker for health outcomes in relationship to the gut microbome. Indeed, chronic inflammation and immune dysregulation resulting from oral bacteria or their products may have systemic effects, which could be associated with intestinal colonization by bacteria and correlated with the cancer health and disease.

There are several mechanisms by which weight gain could contribute to the oral microbiota or vice versa. Many postulate that bacteria in the oral cavity could contribute to systemic metabolic alterations, as with gut Firmicutes. Specific oral taxa could contribute to redirecting consumption of energy by facilitating insulin resistance through increasing levels of TNFα and lipo-polysaccharides or reducing levels of adiponectin. In addition, oral microbiome could also contribute to taste perception^44^ and appetite control.

While our findings were related to the oral cavity, the association between maternal BMI and *Veillonella parvula* in the gut has been previously reported by Costa and colleagues^60^. It is plausible to consider maternal BMI as a proxy variable for child’s diet^61^. In addition to the role of *V. parvula* in association with weight gain, costimulatory properties of *Streptococcus* and *Veillonella* spp. have been observed by several investigators in-vitro and across various human microbial ecosystems^62,63^. Specifically, some streptococci when combined with *Veillonella* substantially augmented immune cell profiles including IL-8, IL-6, IL-10, and TNF-α responses. These data suggest similar interactions and require further investigation particularly in the oral milieu where *Streptococcus* is the predominant genera.

A strength of this study is utilization of high-resolution whole genome sequence data to characterize the oral microbiota. To our knowledge, there is no previous study that has applied shotgun sequencing to saliva samples collected in early childhood for the purpose of this evaluation. Use of 16S data by previous studies lend to poor resolution of oral microbial taxa. Additionally, we were also able to leverage DXA measurements of body composition. To our knowledge, the relationship between DXA-measured fat mass and salivary microbiota has not been explored in children.

Despite these strengths, our findings need to be examined in light of several limitations. The cross-sectional design of this study has its inherent weakness; however, for a subset we were able to address the potential impact of early life growth on development of the oral microbiome. As the main aims of NHBCS did not include dental health assessments, we do not have clinical data on oral diseases and no radiographs were obtained to detect dental caries or bone loss in these child-subjects. While there is clear evidence that oral health status is strongly related to salivary microbiome, our objective was to examine the association between growth and salivary microbiome in the context of stable microbial community and relatively complete dentition. We also recognize that there is the potential for unmeasured or residual confounding based on unexplored associations or due to the use of covariates with dampened effects due to previous, as opposed to, current exposure^41^. Specifically, as discussed earlier, although the impact of diet on the association between weight gain/growth and the establishment of the oral microbiota was not directly assessed in this study, we considered maternal BMI as an alternative indicator and adjusted for it in all our analyses. Other limitations associated with studying the human microbiota in children and the salivary microbiome in particular include the inherent constraints with salivary microbiome characterization (availability of reference databases, frequency and timing of sample collection) and the lack of a priori data for sample size estimation (limiting statistical power to detect differences). Despite these limitations, our data suggests that microbiome-growth assessments using DXA and BMI Z scores were comparable and can be applied to other populations.

In conclusion, our data from 3 to 4-year-old children suggested a lower diversity with increasing BMI and body composition and highlights some differences in oral microbial composition on the basis of BMI (based on overweight status) and TFM. These findings suggest that changes in the body composition might impact the oral microbiome in early childhood or vice versa, further increasing the risk of disease in later life. A larger sample size and prospective follow-up will help determine whether the observed differences become more pronounced as the children grow older, thereby identifying possible mechanisms by which the oral microbiome composition mediates disease. In future analyses, DXA assessment could also be used to explore associations between saliva microbiome and bone remodeling/mass. There is, therefore, need for additional large molecular epidemiologic studies to identify taxonomic and functional links underlying these associations that could be candidates for intervention.

## Methods

### New Hampshire birth cohort study (NHBCS)

The NHBCS is an ongoing prospective cohort study of over 2,250 mother and child dyads from New Hampshire and Vermont, USA. The study was originally designed to assess the long-term effect of arsenic exposure from private well water on children born to pregnant women enrolled at approximately 24 and 28 weeks of gestation^23^. Demographic and anthropometric data were collected via interviews administered prenatally, as well as at multiple timepoints postpartum, and via medical record review. Participants provided written informed consent and all study procedures and protocols were approved by the Center for the Protection of Human Subjects at Dartmouth, and all methods were carried out in accordance with relevant guidelines and regulations. A subset of children with body composition measurements and saliva shotgun sequencing samples processed when the child was 3 or 4 years old were included in this study.

### Childhood growth measurements

Multiple time points and variables were used to describe child growth. In cases when body mass measurements and saliva samples were collected at the same time (within 30 days of each other but generally on the same day), this study design can be considered cross-sectional. For a subset of participants, early life growth measurements (< approximately 2 years of age) were also available and utilized for prospective analyses. Only children that had anthropometric measurements and DXA screening conducted at 3–4 years of age were included in the main analysis assessing associations between body composition and the saliva microbiome cross-sectionally (Supplementary Methods).

*Body mass measurements at 3–4 years*: The anthropometric data were assessed and abstracted from medical records by trained professionals. Data consisted of the following: height (in centimeters) and weight (in kilograms) taken in the clinic during well-child visits when the child was 3 or 4 years of age (1095.75 days < age < 1826.25 days). BMI was analyzed in kg/m^2^ and age- and sex-standardized using the CDC’s child growth charts derived from the package *childsds*^64^ in R. Although we considered CDC’s referenced growth charts to be a better fit for our cohort of US children, we also computed adjusted BMI z-scores using the World Health Organization’s reference charts and found the two methods to be highly correlated (Pearson’s correlation = 99%). At ages 3 and 4, children further underwent a full-body DXA scan to estimate body composition using a Horizon-A Advanced Fan-Beam DXA system (Hologic, Inc; Marlborough, MA, USA) following the protocol from the National Institutes of Health PhenX Toolkit^65^.

Both BMI and DXA measurements were considered to assess child body mass. Although BMI and fat mass both assess child growth, there were a variety of reasons why we explored both in this analysis. We chose to look at BMI because it is a standardized variable that will enable other studies and research groups to compare results with our own. However, as BMI has sensitivity and specificity limitations and DXA has been used as the criterion measure in assessing fat mass in pediatric populations^66,67^, we felt it was also important to consider in this study. Thus, we hypothesize that we may be able to identify some trends across both measurements of child growth but may also find varying associations because BMI reflects both fat and fat-free mass. In summary, using both of these measurements of body mass provides advantages to both internal and external validity.

*Rapid weight gain from birth to age 2 years*: Assessment of RWG was conducted to assess a prospective association between rapid child growth early in life and on saliva microbiome composition. RWG has been associated with increased weight and obesity both later in childhood and into adulthood^29,30^. This score is indicative of the difference between percentile bands on standardized growth charts with 0.67 being the value needed to pass through a centile line^68^. Using delivery and pediatric medical records, we extracted birthweight and a 2-year (+ / 6 months) weight measurement. Children with a gestational age below 37 weeks’ gestation were not included in this analysis to reduce potential confounding from gestational age at birth. Weight-for-age z-scores were calculated using the World Health Organization child growth charts (recommended for clinical use under age 2) and standardized by sex using the *childsds*^64^ in R.

*Growth chart:* In addition to the dichotomous measurement of weight-for-age z-score we modeled growth trajectories of children from birth to 2 years of age using weight-for-length/height ratios. Weight-for-length (or weight-for-height) is recommended as opposed to body mass index in children under 2 years of age^69^. For this analysis, we included children with saliva microbiome samples at 3 or 4 years of age and at least 2 measurements for length and weight before 2 years of age (on or before day 730). Duplicated measurements of weight and height per child were removed and the mean value for the weight-for-length ratio was used if multiple measurements were taken on the same day. Using similar methods to^41^, we used the *fdapace*: Functional Data Analysis and Empirical Dynamics package^70^ in R to create growth trajectories for children. This tool enabled us to build growth curves based on the average value across all children. We used the default settings for the FPCA (functional PCA) command.

### Characterization of the salivary microbiome

Saliva microbiome samples were collected using flocked nylon swabs (Copan Diagnostics) placed in the child’s buccal cavity for 20 s to absorb saliva and placed sponge-down into free conical tubes. Sample collection occurred during the 3-to-4-year study visit that included anthropometric assessment and DXA screening. DNA extractions were performed using the ZymoBiomics Micro-prep kit (Zymo Research). Briefly, tubes containing nylon flocked swab heads were thawed and transferred to ZR BashingBead Lysis Tubes (0.1 & 0.5 mm beads) containing 800 µl Lysis Buffer and 25 µl Proteinase K (20 mg/ml). Lysis tubes were placed in racks in a pre-warmed rotating oven and incubated for 30 min 55 °C and 30 rpm. Bead beating of Lysis tubes was performed in two rounds of 11 min each using a Disruptor Genie (Scientific Industries, Inc.). After lysis, tubes were centrifuged at 10000xg for 30 s in a microcentrifuge to pellet beads. About 400ul of lysate was transferred to Zymo-Spin™ III-F Filter columns and centrifuged at 10000xg for 30 s and collected in a 2 ml collection tube. 1.2 ml of Binding Buffer with 0.5% beta-mercaptoethanol was mixed with the collected lysate and the mixture was centrifuged through Zymo-Spin™ IC Columns for 10000xg for 60 s. Columns were washed with kit provided wash and DNA was eluted in 2 pooled elutions, using each time 19ul of Elution buffer pre-warmed at 60 °C. DNA was quantified using Qubit HS dsDNA kit (Invitrogen) and 2 µl of sample. A yield threshold of 1 ng/µl DNA was required to refer for shotgun sequencing. Above this threshold average DNA yield was 8.6 ± 9.8 ng/µl and ranged from 1 to 57.7 ng/µl. DNA extractions were performed in batches of 12 samples including one external saliva positive control swab and one negative control swab. Average DNA yields of batch positive controls was 15.2 ng/µl and CV of 19%. Negative control yields were too low to quantify at 2 µl. Extracted DNA was amplified from all samples were prepared for sequencing on the NextSeq platform (shotgun metagenomics) using 150 nt paired end reads at the Marine Biological Laboratory (MBL) in Woods Hole, MA using established methods and as previously published^23–25,28^.

All samples were processed as single reads at the MBL and were subsequently processed. First, they underwent quality control to remove contaminants with KneadData v0.7.4. Only saliva samples that had one million reads after KneadData processing were kept in the analysis. Shotgun sequencing samples were functionally profiled using HUMAnN3 version 3.0.0.alpha.3^71^ after being taxonomically profiled using MetaPhlAn3^72^. MetaPhlAn3 and HUMAnN3 jobs were run on Dartmouth’s supercomputer and high-performance computing Linux cluster respectively. HUMAnN3 uses a tiered search approach to first map reads from samples to taxa using marker genes (MetaPhlAn3). Then it creates species-specific pangenomes to provide functional annotations^71^. Only reads from bacteria were considered for this analysis which made up the vast majority (> 99%) of all samples.

### Data analysis

*Covariates:* Covariates were selected based on an a priori literature review. In order to choose which covariates were confounders and needed to be adjusted for in the models, we plotted them on a directed acyclic graph (DAG) (Figure S4). Although our measurement of body mass and saliva microbiome were cross-sectional in our main analysis, we hypothesize a direction of association with body mass as the exposure and the saliva microbiome as the outcome. This was directly examined in our prospective analyses by assessing child growth between 0 and 2 years of age. Based on the DAG, the potential confounders (i.e., related to our exposure and outcome directly or hypothesized to be based on previously identified indirect associations in separate studies) to adjust for in our analyses were age (measured by age in days of the saliva sample), sex (male or female)^24,41^, delivery mode (vaginal or cesarean delivery)^3^, gestational age (age in weeks)^27,28^ and diet (age when child started to eat solid foods in months)^41^. Although previous studies have found associations between maternal BMI or weight gain and children’s BMI^73^, literature identifying associations between maternal BMI and the child’s saliva microbiome have not found associations^41,74^. However, due to previously identified associations between maternal BMI and the child’s gut microbiota^75^, we decided to include maternal BMI measured by self-reported pre-pregnancy weight and measured height (kg per meters squared) as a covariate.

*Descriptive and statistical analyses:* All analyses were completed in R 3.6.0 (http://www.R-project.org). Saliva microbiota sequence read counts were normalized per sample, yielding a compositional relative abundance data set for downstream analyses. We were interested in taxonomic composition at the phylum, genus, and species-level. Each read was classified using the CHOCOPhlAn database within MetaPhlAn3, and, for each sample, ecological diversity measurements (α-diversity—a measure of richness and evenness within a single sample, and β-diversity—a measure of differences between samples) were calculated at the species-level.

To evaluate the relationship between alpha diversity and childhood growth measures, the phyloseq package^76^ was used to compute Shannon diversity. In statistical models, the Shannon alpha diversity was the outcome and was regressed against the relevant exposures using linear regression models. In these models, TFM was transformed into kilograms. Between sample or beta diversity was also assessed using the phyloseq package. Bray–Curtis was used to assess dissimilarity between samples and plotted as a principal coordinate analysis (PCoA) plot to visualize variation. Samples were colored by variables of interest. For overweight status, centroids were plotted with *betadisper* function within the vegan package (ellipses based on 1 standard deviation). Variation between samples was quantified using the adonis2 function from the vegan package^77^ in PERMANOVA analyses. Lastly, Microbiome Multivariable Associations with Linear Models (MaAsLin2)^78^ was used to quantify differences between the relative abundance of taxa by body mass variables adjusted for covariates. MaAsLin2 is specifically designed for ‘omic analyses and uses robust statistical procedures to assess exposures of interest while controlling for other variables. Deviation from default parameters for taxonomic analysis included normalization though the centered-log ratio (CLR) approach and no additional transformation. Interaction variables consider the joint effect of the child growth variable among children in one group (i.e., the joint interaction of female sex and BMI). MaAsLin2 for pathway analysis used default parameters with the additional minimum abundance filtering of 0.0001. Due to the hypothesis-generating and exploratory nature of this study, effect size, p-value, and q-value thresholds were used to determine taxa, genes, and pathways of interest, but thresholds varied by analysis. Thresholds for inclusion in figures are noted in figure legends.

## Supplementary Information

Supplementary Information 1.

Supplementary Information 2 (Table S5- Multiple tabs reflecting MaAsLin2 results).

## Data Availability

The saliva whole metagenomic shotgun sequencing samples are available through the National Center for Biotechnology (NCBI) Sequence Read Archive: https://www.ncbi.nlm.nih.gov/sra (Accession number: PRJNA296814).

## Code availability

The authors will share Rmarkdown scripts upon request.
