# Supplementary material for: Metagenomic analysis reveals associations between salivary microbiota and body composition in early childhood
Source: Sci Rep. 2022 Jul 29;12:13075. doi: 10.1038/s41598-022-14668-y (PMC9338228; doi:10.1038/s41598-022-14668-y)
Supplement: Supplementary file 1 — Supplementary Information 1. [file 41598_2022_14668_MOESM1_ESM.docx]

**Metagenomic Analysis Reveals Associations between Salivary Microbiota and Body Composition in Early Childhood**

Modupe O. Coker^1,2#^, Rebecca M. Lebeaux^1#^, Anne G. Hoen^1^, Yuka Moroishi^1^, Diane Gilbert-Diamond^1^, Erika F. Dade^1^, Juliette C. Madan^1,3^, Margaret R. Karagas^1^

^#^Equal contribution

*Corresponding author

^1^ Department of Epidemiology, Geisel School of Medicine at Dartmouth College, Hanover, NH, 03755, USA

^2^ Department of Oral Biology, School of Dental Medicine, Rutgers, The State University of New Jersey, Newark, NJ, 07103, USA

^3^ Departments of Pediatrics and Psychiatry, Children’s Hospital at Dartmouth, Lebanon, NH, 03766, USA

**Table of Contents**

***Supplementary Figures:***

Figure S1: Sample size overview by exposure assessment and overlap of children by exposure assessment…………………………………………………………………………..2

Figure S2: Correlation between BMI and DXA measured mass at 3-4 years…………....3

Figure S3: Principal coordinate analysis (PCoA) of saliva microbiome samples by sample size and exposure measurements………………………………………………….. 4

Figure S4: Directed acyclic graph (DAG) to assess potential confounders……..…..……5

***Supplementary Tables:***

Table S1: Descriptive overview of children in main cohort by sex (n = 236)……..………6

Table S2: Descriptive overview of children with multiple paired height and weight measurements under 2 years (n = 195)………………………………………..……….…….7

Table S3: Descriptive overview of children by rapid weight gain between 0 and 2 years (n = 157)………………………………………………………………………………….………8

Table S4: Relative abundance (0 to 1) of species by weight status group…………...…..9

Table S5: All MaAsLin2 tables are available as a separate Excel file: table_s5_maaslin2_results_apr6_2022.xlsx

Table S6: Crude PERMANOVA analysis of saliva microbiome samples by exposure group…………………………………………………………………………………………….10

Table S7: Adjusted PERMANOVA analysis of saliva microbiome samples by exposure group………………………………………………………………………………………........11

Table S8: Table comparing 236 children with DXA measurement compared to children excluded………………………………………………………………………………………...12

***Supplementary Methods***……………………………………………………………………13

**A**

**B**

**Figure S1**: Sample size overview by exposure assessment and overlap of children by exposure assessment. **A**) Flow diagram showing breakdown of sample sizes into the main and exploratory cohorts. **B**) Venn diagram showing the number of samples in each cohort and the overlap of samples between cohorts.


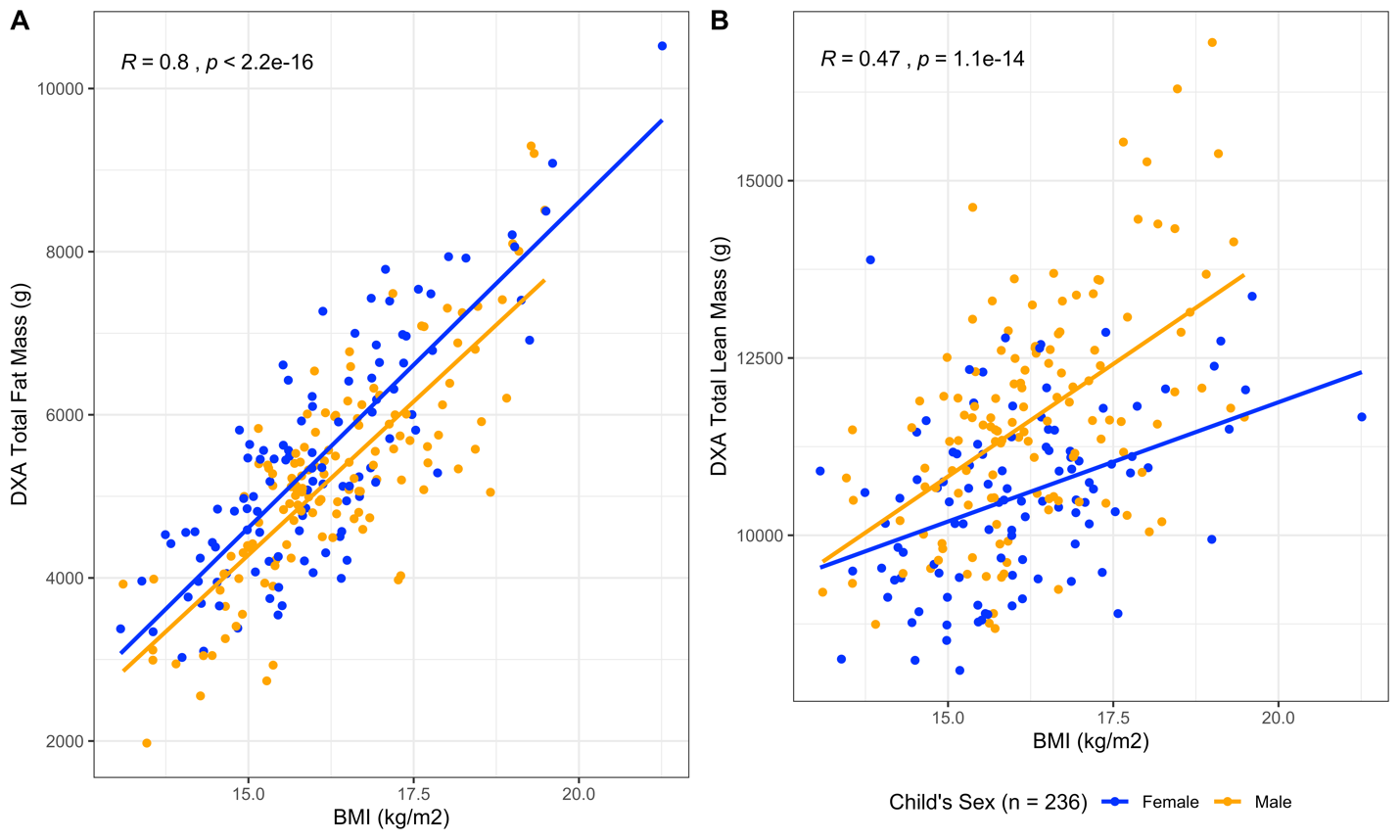
 **Figure S2:** Correlation between BMI and DXA measured mass at 3-4 years. **A**) Correlation of DXA measured total fat mass in grams vs. BMI (kilograms per meter squared). **B**) Correlation of DXA measured total lean mass in grams vs. BMI (kilograms per meter squared). Dots are colored by sex and fit lines are stratified by sex. Pearson correlation was used for overall assessment of association between child growth metrics (unadjusted).

**Figure S3**: Principal coordinate analysis (PCoA) of saliva microbiome samples by sample size and exposure measurements. For all plots, 236 samples were explored and variation was assessed using Bray-Curtis dissimilarity. All child growth metrics were measured at 3-4 years of age. PCoA plot colored by **A**) sample age, **B**) total fat mass measured by DXA scan, **C**) age and sex-adjusted BMI z-score, and **D**) overweight status based on BMI percentile (normal vs. overweight); ellipses were based on 1 standard deviation from the centroid.

**Figure S4:** Directed acyclic graph (DAG) to assess potential confounders.

**Table S1:** Characteristics of 236 children with saliva samples and growth measured at 3 or 4 years.

|  | **Female** | **Male** | **Overall** |
| --- | --- | --- | --- |
|  | **(N=105)** | **(N=131)** | **(N=236)** |
| **Sample age (days)** |  |  |  |
| Mean (SD) | 1400 (84.0) | 1420 (79.9) | 1410 (81.9) |
| Median [Min, Max] | 1380 [1160, 1750] | 1400 [1300, 1680] | 1390 [1160, 1750] |
| **Maternal BMI (kg/m^2^)** |  |  |  |
| Mean (SD) | 25.4 (5.18) | 26.2 (5.44) | 25.8 (5.33) |
| Median [Min, Max] | 24.1 [18.3, 45.7] | 24.9 [17.5, 45.2] | 24.4 [17.5, 45.7] |
| Missing | 3 (2.9%) | 3 (2.3%) | 6 (2.5%) |
| **Delivery method** |  |  |  |
| Vaginal | 77 (73.3%) | 93 (71.0%) | 170 (72.0%) |
| C-section | 27 (25.7%) | 38 (29.0%) | 65 (27.5%) |
| Missing | 1 (1.0%) | 0 (0%) | 1 (0.4%) |
| **Gestational age at birth (weeks)** |  |  |  |
| Mean (SD) | 39.0 (1.92) | 39.0 (1.85) | 39.0 (1.88) |
| Median [Min, Max] | 39.0 [31.8, 42.0] | 39.3 [31.0, 43.0] | 39.1 [31.0, 43.0] |
| **Solid foods start age (months)** |  |  |  |
| Mean (SD) | 5.46 (1.25) | 5.14 (1.32) | 5.28 (1.30) |
| Median [Min, Max] | 6.00 [1.00, 9.00] | 5.00 [2.00, 10.0] | 5.00 [1.00, 10.0] |
| Missing | 17 (16.2%) | 13 (9.9%) | 30 (12.7%) |
| **Body Mass Index (kg/m^2^)** |  |  |  |
| Mean (SD) | 16.0 (1.47) | 16.3 (1.34) | 16.2 (1.40) |
| Median [Min, Max] | 15.9 [13.1, 21.3] | 16.1 [13.1, 19.5] | 16.0 [13.1, 21.3] |
| **Weight status based on BMI percentiles (CDC)** |  |  |  |
| Underweight | 3 (2.9%) | 6 (4.6%) | 9 (3.8%) |
| Normal | 75 (71.4%) | 90 (68.7%) | 165 (69.9%) |
| Overweight | 19 (18.1%) | 16 (12.2%) | 35 (14.8%) |
| Obese | 8 (7.6%) | 19 (14.5%) | 27 (11.4%) |
| **Height (cm)** |  |  |  |
| Mean (SD) | 101 (4.00) | 103 (4.27) | 102 (4.26) |
| Median [Min, Max] | 100 [91.2, 117] | 103 [92.9, 115] | 102 [91.2, 117] |
| **Weight (kg)** |  |  |  |
| Mean (SD) | 16.3 (2.07) | 17.3 (2.32) | 16.8 (2.26) |
| Median [Min, Max] | 16.2 [12.7, 22.4] | 17.2 [12.0, 25.0] | 16.7 [12.0, 25.0] |
| **Total fat mass (g)** |  |  |  |
| Mean (SD) | 5440 (1420) | 5240 (1260) | 5330 (1340) |
| Median [Min, Max] | 5190 [3030, 10500] | 5200 [1970, 9300] | 5200 [1970, 10500] |
| **Total lean mass (g)** |  |  |  |
| Mean (SD) | 10500 (1210) | 11600 (1590) | 11200 (1530) |
| Median [Min, Max] | 10500 [8090, 13900] | 11600 [8690, 16900] | 11100 [8090, 16900] |

**Table S2**: Descriptive overview of children with multiple paired height and weight measurements under 2 years (n = 195).

|  | **Female** | **Male** | **Overall** |
| --- | --- | --- | --- |
|  | **(N=89)** | **(N=106)** | **(N=195)** |
| **Sample age (days)** |  |  |  |
| Mean (SD) | 1400 (85.1) | 1410 (84.9) | 1410 (85.0) |
| Median [Min, Max] | 1380 [1310, 1750] | 1380 [1310, 1680] | 1380 [1310, 1750] |
| **Maternal BMI (kg/m^2^)** |  |  |  |
| Mean (SD) | 26.1 (5.78) | 25.9 (5.08) | 26.0 (5.39) |
| Median [Min, Max] | 24.3 [18.3, 45.7] | 24.7 [17.5, 45.2] | 24.5 [17.5, 45.7] |
| Missing | 3 (3.4%) | 2 (1.9%) | 5 (2.6%) |
| **Delivery method** |  |  |  |
| Vaginal | 68 (76.4%) | 77 (72.6%) | 145 (74.4%) |
| Delivery | 21 (23.6%) | 29 (27.4%) | 50 (25.6%) |
| **Gestational age at birth (weeks)** |  |  |  |
| Mean (SD) | 38.9 (1.80) | 39.0 (1.87) | 39.0 (1.84) |
| Median [Min, Max] | 39.0 [31.8, 42.0] | 39.3 [29.1, 43.0] | 39.1 [29.1, 43.0] |
| **Solid foods start age (months)** |  |  |  |
| Mean (SD) | 5.31 (1.32) | 5.20 (1.19) | 5.25 (1.25) |
| Median [Min, Max] | 5.25 [1.00, 9.00] | 5.00 [3.00, 10.0] | 5.00 [1.00, 10.0] |
| Missing | 15 (16.9%) | 8 (7.5%) | 23 (11.8%) |
| **Body Mass Index (kg/m^2^)** |  |  |  |
| Mean (SD) | 16.2 (1.58) | 16.3 (1.39) | 16.2 (1.48) |
| Median [Min, Max] | 16.0 [13.1, 21.3] | 16.1 [13.5, 19.5] | 16.0 [13.1, 21.3] |
| **Weight status based on BMI percentiles (CDC)** |  |  |  |
| Underweight | 3 (3.4%) | 5 (4.7%) | 8 (4.1%) |
| Normal | 62 (69.7%) | 72 (67.9%) | 134 (68.7%) |
| Overweight | 14 (15.7%) | 14 (13.2%) | 28 (14.4%) |
| Obese | 10 (11.2%) | 15 (14.2%) | 25 (12.8%) |
| **Height (cm)** |  |  |  |
| Mean (SD) | 101 (4.23) | 102 (4.62) | 102 (4.50) |
| Median [Min, Max] | 101 [91.2, 117] | 103 [87.7, 115] | 102 [87.7, 117] |
| **Weight (kg)** |  |  |  |
| Mean (SD) | 16.5 (2.23) | 17.1 (2.51) | 16.8 (2.41) |
| Median [Min, Max] | 16.2 [12.2, 22.4] | 17.0 [11.9, 25.0] | 16.7 [11.9, 25.0] |
| **Total fat mass (g)** |  |  |  |
| Mean (SD) | 5430 (1450) | 5210 (1370) | 5310 (1410) |
| Median [Min, Max] | 5170 [3340, 10500] | 5100 [1970, 9300] | 5120 [1970, 10500] |
| Missing | 15 (16.9%) | 11 (10.4%) | 26 (13.3%) |
| **Total lean mass (g)** |  |  |  |
| Mean (SD) | 10600 (1290) | 11700 (1660) | 11200 (1600) |
| Median [Min, Max] | 10700 [8090, 13900] | 11600 [8740, 16900] | 11200 [8090, 16900] |
| Missing | 15 (16.9%) | 11 (10.4%) | 26 (13.3%) |

| **Table S3:** Descriptive overview of children by rapid weight gain between 0 and 2 years (n = 157) | **Not rapid growth** | **Rapid growth** | **Overall** |
| --- | --- | --- | --- |
|  | **(N = 112)** | **(N = 45)** | **(N = 157)** |
| **Sample age (days)** |  |  |  |
| Mean (SD) | 1410 (82.9) | 1400 (82.2) | 1410 (82.6) |
| Median [Min, Max] | 1390 [1310, 1630] | 1370 [1310, 1680] | 1380 [1310, 1680] |
| **Sex** |  |  |  |
| Female | 49 (43.8%) | 26 (57.8%) | 75 (47.8%) |
| Male | 63 (56.2%) | 19 (42.2%) | 82 (52.2%) |
| **Maternal BMI (kg/m^2^)** |  |  |  |
| Mean (SD) | 26.2 (5.07) | 26.4 (6.03) | 26.3 (5.35) |
| Median [Min, Max] | 24.9 [18.3, 43.4] | 25.2 [18.3, 45.7] | 24.9 [18.3, 45.7] |
| Missing | 3 (2.7%) | 1 (2.2%) | 4 (2.5%) |
| **Delivery method** |  |  |  |
| Vaginal | 88 (78.6%) | 34 (75.6%) | 122 (77.7%) |
| C-section | 24 (21.4%) | 11 (24.4%) | 35 (22.3%) |
| **Gestational age at birth (weeks)** |  |  |  |
| Mean (SD) | 39.7 (1.11) | 38.9 (1.23) | 39.5 (1.19) |
| Median [Min, Max] | 39.9 [37.0, 42.0] | 39.0 [37.0, 41.0] | 39.6 [37.0, 42.0] |
| **Solid foods start age (months)** |  |  |  |
| Mean (SD) | 5.25 (1.21) | 5.10 (1.31) | 5.21 (1.23) |
| Median [Min, Max] | 5.00 [3.00, 10.0] | 5.00 [1.00, 8.00] | 5.00 [1.00, 10.0] |
| Missing | 10 (8.9%) | 6 (13.3%) | 16 (10.2%) |
| **Body Mass Index (kg/m^2^) at 3-4 years** |  |  |  |
| Mean (SD) | 15.9 (1.30) | 17.0 (1.66) | 16.2 (1.49) |
| Median [Min, Max] | 15.8 [13.1, 19.6] | 16.6 [13.6, 21.3] | 16.0 [13.1, 21.3] |
| **Weight status based on BMI percentiles (CDC) at 3-4 years** |  |  |  |
| Underweight | 6 (5.4%) | 1 (2.2%) | 7 (4.5%) |
| Normal weight | 86 (76.8%) | 25 (55.6%) | 111 (70.7%) |
| Overweight | 11 (9.8%) | 7 (15.6%) | 18 (11.5%) |
| Obese | 9 (8.0%) | 12 (26.7%) | 21 (13.4%) |
| **Height (cm) at 3-4 years** |  |  |  |
| Mean (SD) | 102 (4.58) | 103 (3.77) | 102 (4.38) |
| Median [Min, Max] | 102 [91.2, 117] | 103 [94.1, 115] | 102 [91.2, 117] |
| **Weight (kg) at 3-4 years** |  |  |  |
| Mean (SD) | 16.5 (2.20) | 18.0 (2.47) | 17.0 (2.37) |
| Median [Min, Max] | 16.4 [12.0, 24.1] | 17.7 [13.1, 25.0] | 16.8 [12.0, 25.0] |
| **Total fat mass (g) at 3-4 years** |  |  |  |
| Mean (SD) | 5110 (1190) | 5970 (1550) | 5350 (1350) |
| Median [Min, Max] | 5090 [2930, 9080] | 5580 [3340, 10500] | 5130 [2930, 10500] |
| Missing | 14 (12.5%) | 6 (13.3%) | 20 (12.7%) |
| **Total lean mass (g) at 3-4 years** |  |  |  |
| Mean (SD) | 11100 (1620) | 11700 (1640) | 11300 (1640) |
| Median [Min, Max] | 11200 [8090, 16300] | 11600 [9100, 16900] | 11300 [8090, 16900] |
| Missing | 14 (12.5%) | 6 (13.3%) | 20 (12.7%) |

**Table S4:** Relative abundance (0 to 1) of species by weight status group. To match **Figure 2**, only the top 10 species with the greatest mean abundance across all groups are shown.

| **Species with highest relative abundance across all samples** | **Under weight** | **Normal** | **Overweight** | **Obese** |
| --- | --- | --- | --- | --- |
| *Gemella haemolysans* | 0.08878074 | 0.11703323 | 0.12813775 | 0.12299794 |
| *Granulicatella elegans* | 0.01464825 | 0.03216753 | 0.03100268 | 0.03307133 |
| *Neisseria flavescens* | 0.07245013 | 0.05374267 | 0.0509048 | 0.04555302 |
| *Neisseria sicca* | 0.03603204 | 0.03064603 | 0.03091344 | 0.03420358 |
| *Rothia mucilaginosa* | 0.05917168 | 0.05886365 | 0.04383201 | 0.04878143 |
| *Streptococcus infantis* | 0.03008863 | 0.02805165 | 0.02308923 | 0.02045325 |
| *Streptococcus mitis* | 0.13493933 | 0.23860259 | 0.26348249 | 0.28484386 |
| *Streptococcus oralis* | 0.04002418 | 0.03052285 | 0.03172552 | 0.0262107 |
| *Streptococcus salivarius* | 0.0646391 | 0.03986259 | 0.02982987 | 0.03972381 |
| *Streptococcus sanguinis* | 0.03263516 | 0.04295001 | 0.03852994 | 0.03507568 |

**Table S5:** MaAsLin2 tables available as an additional Supplementary file with separate tabs.

**Table S6:** Crude PERMANOVA analysis of saliva microbiome samples by exposure group. PERMANOVA analysis of 1000 permutations to assess if child growth metrics contribute to the variation across 236 saliva microbiome samples measured by Bray-Curtis dissimilarity metrics at the species-level.

| **Model: Total fat mass measured by DXA scan** | | | | | |
| --- | --- | --- | --- | --- | --- |
|  | Degrees of freedom | Sum of squares | R^2^ | F | Pr(>F) |
| Total fat mass DXA in grams | 1 | 0.400 | 0.00423 | 2.8951 | 0.008991 |
| Residual | 234 | 32.321 | 0.98778 |  |  |
| Total | 235 | 32.721 | 1.0000 |  |  |
| **Model: Age and sex-adjusted BMI z-score** | | | | | |
| BMI z-score | 1 | 0.341 | 0.01041 | 2.4616 | 0.02098 |
| Residual | 234 | 32.380 | 0.98959 |  |  |
| Total | 235 | 32.721 | 1.0000 |  |  |
| **Model: Overweight status** | | | | | |
| Overweight | 1 | 0.187 | 0.0057 | 1.3415 | 0.1998 |
| Residual | 234 | 32.535 | 0.9943 |  |  |
| Total | 235 | 32.721 | 1.0000 |  |  |

**Table S7:** Adjusted PERMANOVA analysis of saliva microbiome samples by exposure group. PERMANOVA analysis of 1000 permutations to assess whether child growth metrics and covariates contribution to the variation across 202 saliva microbiome samples measured by Bray-Curtis dissimilarity metrics at the species-level.

| **Model: Total fat mass measured by DXA scan** | | | | | |
| --- | --- | --- | --- | --- | --- |
|  | Degrees of freedom | Sum of squares | R^2^ | F | Pr(>F) |
| Total fat mass DXA in grams | 1 | 0.3114 | 0.01114 | 2.2766 | 0.03197 |
| Sample age in days | 1 | 0.2264 | 0.00810 | 1.6550 | 0.11089 |
| Male | 1 | 0.2180 | 0.00780 | 1.5939 | 0.10490 |
| Solid foods start age in months | 1 | 0.0695 | 0.00249 | 0.5083 | 0.85115 |
| Gestational age in weeks | 1 | 0.2068 | 0.00740 | 1.5115 | 0.14186 |
| Maternal BMI (kg/m2) | 1 | 0.2483 | 0.00889 | 1.8155 | 0.08492 |
| C-section | 1 | 0.1257 | 0.00450 | 0.9187 | 0.43856 |
| Residual | 194 | 26.5378 | 0.94968 |  |  |
| Total | 201 | 27.9440 | 1.000 |  |  |
| **Model: Age and sex-adjusted BMI z-score** | | | | | |
| BMI z-score | 1 | 0.2318 | 0.00830 | 1.6916 | 0.08791 |
| Sample age in days | 1 | 0.2322 | 0.00831 | 1.6941 | 0.10889 |
| Male | 1 | 0.2260 | 0.00809 | 1.6493 | 0.09391 |
| Solid foods start age in months | 1 | 0.0727 | 0.00260 | 0.5302 | 0.85315 |
| Gestational age in weeks | 1 | 0.2013 | 0.00720 | 1.4690 | 0.14486 |
| Maternal BMI (kg/m2) | 1 | 0.2706 | 0.00968 | 1.9749 | 0.05894 |
| C-section | 1 | 0.1240 | 0.00444 | 0.9046 | 0.49251 |
| Residual | 194 | 26.5854 | 0.95138 |  |  |
| Total | 201 | 27.9440 | 1.000 |  |  |
| **Model: Overweight status** | | | | | |
| Overweight status | 1 | 0.1369 | 0.00490 | 0.9940 | 0.3766 |
| Sample age in days | 1 | 0.2350 | 0.00841 | 1.7071 | 0.0959 |
| Male | 1 | 0.2256 | 0.00807 | 1.6385 | 0.1009 |
| Solid foods start age in months | 1 | 0.0771 | 0.00276 | 0.5596 | 0.8262 |
| Gestational age in weeks | 1 | 0.1963 | 0.00703 | 1.4259 | 0.1658 |
| Maternal BMI (kg/m2) | 1 | 0.2377 | 0.00851 | 1.7268 | 0.0989 |
| C-section | 1 | 0.1253 | 0.00449 | 0.9103 | 0.4545 |
| Residual | 194 | 26.7100 | 0.95584 |  |  |
| Total | 201 | 27.9440 | 1.000 |  |  |

**Table S8:** Table comparing 236 children with DXA measurement compared to children excluded.

|  | **Excluded**  **(n = 37)** | **Included**  **(n = 236)** | **Overall**  **(n = 273)** | **P*-*value*** |
| --- | --- | --- | --- | --- |
| **Sample age (in days)** |  |  |  | 0.02* |
| Mean (SD) | 1380 (50.9) | 1410 (81.9) | 1400 (79.1) |  |
| Median [Min, Max] | 1360 [1320, 1500] | 1390 [1160, 1750] | 1380 [1160, 1750] |  |
| **Infant's sex** |  |  |  | 1 |
| Female | 17 (45.9%) | 105 (44.5%) | 122 (44.7%) |  |
| Male | 20 (54.1%) | 131 (55.5%) | 151 (55.3%) |  |
| **Maternal BMI (kg/m^2^)** |  |  |  | 0.32 |
| Mean (SD) | 27.2 (6.24) | 25.8 (5.33) | 26.0 (5.48) |  |
| Median [Min, Max] | 25.0 [20.0, 43.4] | 24.4 [17.5, 45.7] | 24.6 [17.5, 45.7] |  |
| Missing | 0 (0%) | 6 (2.5%) | 6 (2.2%) |  |
| **Delivery method** |  |  |  | 0.82 |
| Vaginal | 28 (75.7%) | 170 (72.0%) | 198 (72.5%) |  |
| C-section | 9 (24.3%) | 65 (27.5%) | 74 (27.1%) |  |
| Missing | 0 (0%) | 1 (0.4%) | 1 (0.4%) |  |
| **Gestational age (in weeks)** |  |  |  | 0.70 |
| Mean (SD) | 39.0 (2.16) | 39.0 (1.88) | 39.0 (1.92) |  |
| Median [Min, Max] | 39.4 [29.1, 42.0] | 39.1 [31.0, 43.0] | 39.1 [29.1, 43.0] |  |
| **Solid foods start age (in months)** |  |  |  | 0.14 |
| Mean (SD) | 4.91 (1.15) | 5.28 (1.30) | 5.23 (1.28) |  |
| Median [Min, Max] | 5.00 [2.00, 7.00] | 5.00 [1.00, 10.0] | 5.00 [1.00, 10.0] |  |
| Missing | 5 (13.5%) | 30 (12.7%) | 35 (12.8%) |  |
| **BMI (kg/m^2^)** |  |  |  | 0.16 |
| Mean (SD) | 16.5 (1.39) | 16.2 (1.40) | 16.2 (1.41) |  |
| Median [Min, Max] | 16.3 [13.8, 20.0] | 16.0 [13.1, 21.3] | 16.0 [13.1, 21.3] |  |
| **Weight status CDC** |  |  |  | 0.23 |
| Normal | 22 (59.5%) | 165 (69.9%) | 187 (68.5%) |  |
| Obese | 6 (16.2%) | 27 (11.4%) | 33 (12.1%) |  |
| Overweight | 9 (24.3%) | 35 (14.8%) | 44 (16.1%) |  |
| Underweight | 0 (0%) | 9 (3.8%) | 9 (3.3%) |  |
| **Height (in cm)** |  |  |  | < 0.01* |
| Mean (SD) | 99.7 (4.32) | 102 (4.26) | 102 (4.33) |  |
| Median [Min, Max] | 99.8 [87.7, 109] | 102 [91.2, 117] | 102 [87.7, 117] |  |
| **Weight (in kg)** |  |  |  | 0.53 |
| Mean (SD) | 16.5 (2.25) | 16.8 (2.26) | 16.8 (2.26) |  |
| Median [Min, Max] | 16.7 [11.9, 21.6] | 16.7 [12.0, 25.0] | 16.7 [11.9, 25.0] |  |

*Statistically significant at p < 0.05. P*-*value measured using Kruskal-Wallis for continuous variables and the chi-square test for categorical variables.

**Supplementary Methods:**

Out of the 273 children in this study with weight and height measurement in addition to a saliva shotgun sequencing sample at 3 or 4 years of age, 237 children had DXA profiling available. Only children that had height/weight measurements within the same month (i.e., <30 days apart) of the DXA measurement were considered for this study. This restriction only removed one infant creating our final sample size of 236. To assess if there were any differences between children with and without DXA measurements, we assessed demographic characteristics between the 37 children not included in the study with the 236 children that were. The only variables that were different between the two groups were age of sample collection and height which were both higher in the groups exposed to DXA [see **Table S8** above]. While the differences were statistically significantly different, we don’t believe they denoted a strong bias other than the children were slightly older among children who received DXA. This makes sense as children who are too young may struggle to hold still for the duration of the DXA scan.
